# Orthodontia

**Published:** 1888-03

**Authors:** N. S. Hoff


### Orthodontia.

BY N. S. HOFF, D.D.S.

(Read before the Odontological Society of Cincinnati, Nov. 22, 1887.)

Probably the highest type of physical beauty to which the human race has ever attained, was that of the ancient Greeks and Romans. But this beautiful and symmetrical physique was not attained by allowing nature to do the best it could, while laws of health and hygiene were totally disregarded, and the pleasures and dissipations of fashion and society were indulged in the regardless manner in our day and generation. Among these people the body was well thought of, and much care was taken to keep it in the best possible condition. Their pictures do not show deformed jaws or indications of disorganized sets of teeth, and we do not know that dental interference on the part of the surgeon was at all common. Is it not possible that dental derangements belong more especially to our time because of the high state of excitement and worry and consequent bad health induced by dissipation and disregard of health laws, than to an advanced civilization?

This, by way of an introduction to a few thoughts that we wish to present concerning one of the most unbecoming maladies which we as dentists are called upon to treat, and which we also believe is largely the result of the infraction of the common laws of health and decency. While the prevailing style of dressing children may not be altogether indecent, it certainly is in the light of other habits and customs not healthy. The children of to-day live too much in the house, and when sent on the street, they are dressed for the ball room. We have often wondered how the poor little things could endure the severe weather and grow. In fact they do not grow as nature intended they should, else we would not be here to discuss such a question as irregularities of the teeth, one of the most unsightly of all ordinary deformities.

We look upon most deformities of the human body as something terrible, and consider no effort made for their relief untimely or too expensive. Yet the very large majority of our patients are more or less afflicted with irregularity of their teeth in some form or degree, and we do little or nothing by way of advice or professional interference to prevent or relieve these conditions, and sometimes by unskillful operations or unwise extractions do much to produce such conditions. It is also true that we oftentimes ignore these conditions, preferring not to undertake their treatment; unless the patient or interested friends, demand some interference, generally, because of the practical difficulties in the way of accomplishing any thing that will be of permanent benefit; or from lack of pecuniary compensation. But we think the reason why more has not been done in this way by the profession is because of the lack of definite knowledge as to conditions and methods of procedure from well established scientific principles. We are persuaded on seeing these cases of malformed teeth and jaws, and irregular positions of the teeth, that at some time during the previous life of our patient, or some of his ancestry, interfence of some sort has taken place to change the systematic and perfect plan of nature to supply such organs as shall be required to perform the functions necessary to the maintenance of a perfect human economy.

These causes are usually classified under two general heads as *Hereditary and Acquired.*

Hereditary cases are exemplified in deformities already existing in the parents, being transmitted to their children. And again in children of parents of widely different form or extent of bone development, resulting in conflicting tendencies in the natural type of the child, for example a large jaw and small teeth, or a small jaw and large teeth.

Acquired deformities are more numerous, and are those that we have been most accustomed to think susceptible of treatment, although their causes are more difficult to determine. Some of the most prominent causes are said to be the premature loss or too long retention of the deciduous teeth, accident, improper habit, delayed development or eruption of one or more teeth, unusual or excessive development of the soft tissues of the face, mouth, and throat, defective nutrition and general bad systemic condition by folly, dissipation, etc.

It is evident from the above that some thing has been done in the way of determining the causes of this condition, and we are also aware that much is constantly being done in the way of treatment. Whether this malady is becoming more common, or the public more intelligent and sensitive in regard to it, or the horizon of our professional field is enlarging, we are not prepared to state, but it is a fact that in our experience there seems to be a growing necessity for treatment of this condition, which not unfrequently is in the shape of a demand on the part of the victim himself. Generally when patients demand it, it is for relief from distorted conditions of the face or mouth, and they seem only to desire to have a comely face put on them; seemingly with no appreciation of the fact that a perfect occlusion of the teeth is more desirable as a health producer and a consequent beautifyer as well. But these obtrusive forms of this malady are by no means the most hurtful if they are the most unsightly. A careful scrutiny of the mouths which we are called upon from day to day to operate in, will reveal an astonishingly large per centage of badly adjusted sets of teeth and very few perfect occlusions. In almost every adult we will find one or more teeth missing, and as a censequence, a broken antagonism. Constant and long use generally makes an occlusion between opposing or adjoining teeth that renders mastication possible—though necessarily defective—and allows comfortable rest and support to the jaw. But these forced articulations are almost certain to render the natural and free motions of the jaw impossible, and usually the teeth are twisted and crowded out of their natural positions, producing inviting and commodious apartments for the production and lodgement of whole families of bacteria and other undesirable disease producing tenants. This form of acquired irregularity of the teeth we regard as more detrimental to the best health, if not to the highest type of beauty, than any other.

With this general summary by way of recognition of this affliction, for it assuredly is an affliction, leading as it does to such destructive and painful results, we desire to suggest a few ideas that may be of practical use, in our endeavors as dentists, to meet this affliction with an adequate understanding of its nature, including cause, and be prepared to give preventive as well as remedial advice, not only when called upon for such advice, but also whenever in the full recognition of our duty to patients we know such advice is needed. In short making the conservation of a perfect antagonism of the teeth of no less moment than the existence of any particular tooth.

And we believe that the need is not specially urgent for more perfect appliances with which the crooks and snarls may be taken out of a badly deranged set of teeth as if by magic and with little or no care or anxiety for patient or operator, but for more systematic and definite knowledge of what constitutes a perfect dentition, and the general and specific principles that govern its development and operation. This would involve concentrated study, not so much of conditions or peculiarities of irregular dentition, as careful comparative anatomical study, for the purpose of establishing a type or form of the human jaw that is natural and beautiful, and such anatomical, histological, physiological, pathological, and philosophical study as may be necessary to clearly indicate the location, constitution, construction, function, and peculiarities of the several organs and tissues which may be involved in the treatment of this malady, supplementing such study with a classification of reported cases in practice, with full and complete statistics, including models, drawings, pictures, or descriptions, of every condition or circumstance in any way influencing the perpetuation of the malady, or rendering it more susceptible or difficult of treatment, making a record of any circumstance or fact that would be of service in establishing the truth or falsity of a theory beyond question. In this way only could we have a well established scientific theory, for it would have science as well as experiment for a basis, for the treatment of all disordered conditions of the dental organs.

In this suggestion we are not unmindful of all that has been done by a few noble earnest men in our profession in the past, but it is the very fact that something has already been achieved in this direction that suggests the probability that more may be known if more concentrated effort was directed to the accomplishment of this object. At least what we have now as statements from individuals might be confirmed or disproved by more careful and extended research, and what we now know theoretically may be solidified into absolute laws or at least well founded principles, which would be of incalculable service in determining proper methods for procedure in the conduct of the varied and troublesome cases constantly presenting for treatment.

In most other departments of our profession the day of experiment is passed and methods of procedure have settled down upon well established theories. While many cases in practice have been described in our dental literature of the irregularities of the teeth, very little has been said about general principles, and that little has come forth so timidly and meagerly as to inspire little or no confidence, except as the theory of one idea. Now, what we would suggest would be that some competent persons undertake to establish general principles of theory and practice, founded upon ample scientific investigation, that should command the confidence of the profession. We also believe such knowledge is possible and practicable.

In conclusion we would suggest that this work might, with much profit and probably great honor, be undertaken by this society or such members as will agree to carry on a certain portion that they may elect, to a satisfactory conclusion, even though it may take years to arrive at any practical results, and we are certain that none would accuse us of "hunting a rut to run in," but when it was known that one society was thus organized for definite and special work, others would be stimulated and induced to enter in a similar manner the investigation of other important departments of our profession, and before long we would be organized into one grand university with no local habitation save the Dental Scientific World, and the progress of our profession would continue to outrun all others in the future as in the past few years it has, in scientific attainments.

